# Countermovement Jump Analysis as a Predictor of Overhead Pitching Velocity in Adolescent Baseball Pitchers

**DOI:** 10.5114/jhk/211720

**Published:** 2025-11-19

**Authors:** Hui-Wen Hsiao, Heng-Ju Lee

**Affiliations:** 1 Department of Physical Education and Sports Sciences, National Taiwan Normal University, Taipei, Taiwan.

**Keywords:** ball speed, ground reaction forces, lower extremity, performance evaluation

## Abstract

Lower limb strength is crucial in pitching, as force output correlates positively with pitching velocity. The countermovement jump (CMJ) is a common test for assessing lower limb strength, and previous research has hinted at its potential to predict performance in adult baseball players. However, further research is warranted concerning adolescent players. This study investigated the relationship between CMJ lower limb power and fastball velocity in adolescent baseball pitchers. Thirty-two adolescent male baseball pitchers from junior high school baseball teams executed three CMJs and threw five fastballs from a custom-made pitching mound. A Kistler force plate (2500 Hz) recorded ground reaction forces (GRFs) during the CMJ, and a pocket radar gun measured ball velocity. The Pearson correlation coefficient assessed the relationship between CMJ variables and ball speed. A stepwise-forward multiple regression model determined the contribution of CMJ variables to predicting fastball velocity in adolescent baseball pitchers. The CMJ variables (braking force, peak force, braking rate of force development, braking impulse, propulsive impulse, peak power, rate of power development, and leg stiffness) correlated positively with ball velocity. The regression analysis revealed that absolute braking force and body height explained 43.3% of the variance in velocity. In conclusion, adolescent pitchers with higher absolute braking force during the CMJ and greater body height are likely to achieve higher fastball velocity. Lower limb strength, assessed via CMJ tests, may aid in predicting adolescent pitchers' performance. These findings emphasize the importance of lower limb strength for pitching velocity and provide guidance to coaches for developing training programs to enhance adolescent players’ fastball velocity.

## Introduction

In baseball, pitching is a complex and dynamic task, and pitching velocity is a key performance indicator that has been studied extensively. The primary emphasis in pitching biomechanics research centers on the upper arm, owing to its pivotal role in generating the highest angular velocities and joint moments during the pitching cycle. However, pitchers perform pitching and transfer ground reaction forces through the kinetic chain, which provides neuromuscular functions with appropriate activation and coordination (Kibler et al., 2013; Kibler and Sciascia, 2004; Seroyer et al., 2010). The lower limbs and the trunk have been reported to contribute 51–55% of the kinetic energy transmitted to the hand during pitching (Lintner et al., 2008). During the initial pitching motion, the initial kinetic chain transfers energy and forces through the lower limb from the proximal to distal segments, influencing the stress and risk of arm injury (Aguinaldo and Escamilla, 2019; Aguinaldo et al., 2007; Bullock et al., 2022; Deal et al., 2020) and performance (Howenstein et al., 2019, 2020; Palmer et al., 2015). Therefore, the lower extremities are essential to provide a foundation for pitching.

Numerous studies have examined the effect of lower-body pitching mechanics on stride leg ground reaction force and its relationship with pitching velocity (Guido and Werner, 2012; Kageyama et al., 2014; MacWilliams et al., 1998; Manzi et al., 2021; McNally et al., 2015; Werner et al., 2008). Studies have suggested that the strength of the stride leg muscles assists with bracing and braking forces, which should contribute to better force transfer and help pitchers achieve the maximum pitch velocity (Campbell et al., 2010; Manzi et al., 2023; Matsuo et al., 2001). In addition, earlier research found a positive and significant correlation between the amount of force generated by the lower limbs during the pitching motion and pitching velocity (Howenstein et al., 2020). Stride leg forces during pitching are strong predictors of ball velocity (McNally et al., 2015), demonstrating that strength and capability of the lower-body muscle are essential for pitching mechanics (Howenstein et al., 2020; Sakurai et al., 2023). Therefore, assessing lower-extremity neuromuscular function may help determine the injury risk and athletic performance in baseball players.

In addition, the countermovement jump (CMJ) has been identified as a common, effective, and simple test for evaluating athletic performance by collecting physical performance data. It has not only been shown to be useful for monitoring neuromuscular status (Claudino et al., 2017), but has also been used to collect physical performance data to assess athletic performance and lower extremity power (Mangine et al., 2013a; Markovic et al., 2004; Moir et al., 2004; Nuzzo et al., 2008). The analysis of different phases of the CMJ can be used to distinguish between athletes’ competitive levels (Suárez-Balsera et al., 2025). As a predictor of baseball-specific performance, lower-limb power performance from the vertical jump has been shown to predict batting and base run performance during a professional baseball season (Hoffman et al., 2009; Mangine et al., 2013b; Teske et al., 2021). Force development in the lower extremities plays a significant role in both the performance and injury prevention during pitching. Recent studies have also demonstrated a significant correlation between the summation of CMJ variables such as peak force, peak power, the rate of power development, jump height, and ball spin in professional baseball pitchers (Wong et al., 2023). Mayberry et al. (2020) investigated the relationship between the results of lower-body testing and the risk of throwing-related arm injuries by measuring the CMJ in professional baseball pitchers. According to that research, players who had lower rates of force development during the eccentric phase and uneven force output during the concentric phase of their jump were more likely to have a higher risk of elbow injury during their career (Mayberry et al., 2020).

Emphasis on sports performance testing has recently increased. While previous studies have focused primarily on professional baseball pitchers, there is a gap in the knowledge regarding the relationship between jump performance, specifically the countermovement jump, and baseball pitching performance in adolescent players. Lower limb strength required for pitchers to pitch effectively may reflect the force-delivered patterns in CMJ tests. Further research is required to better understand the validity and application of jump tests in the assessment of baseball pitchers (Sakurai et al., 2023). Development-related changes in performance and physical characteristics should be considered in teenagers (Nakata et al., 2013). Therefore, it is unclear whether CMJ tests can be considered valid assessments of a teenager's pitching performance potential. Further research is needed to investigate the link between CMJ scores and pitching ability among adolescent baseball pitchers.

This study had two main objectives. The first was to examine the relationship between anthropometric measurements, CMJ performance variables, and fastball velocity in adolescent baseball pitchers. The second was to determine the contribution of the overall CMJ variables in predicting fastball velocity in this population. We hypothesized that adolescent baseball pitchers who demonstrated higher levels of lower-extremity force and better CMJ performance would exhibit greater fastball velocity. Additionally, we hypothesized that certain CMJ variables would help explain the variance in predicting the fastball velocity in this population.

## Methods

### Participants

Thirty-two teenage male baseball pitchers were recruited from junior high school baseball teams. Their mean age was 14.1 ± 0.5 years, with an average body height of 1.73 ± 0.05 m and body mass of 66.1 ± 12.5 kg. All participants had played baseball for their school teams for at least two years and had prior pitching mound experience in games. Participants were deemed healthy with no injuries or pain during the examination. Participants were excluded if they had any recent injury history that prevented them from taking part in sports training within the past six months, reported pain during training or pitching, or had a history of orthopedic surgeries on their throwing arm or lower extremity. The study was approved by the Institutional Review Board of the National Taiwan Normal University, Taipei, Taiwan (approval code: 201912HM108; approval date: 03 July 2020), and written informed consent was obtained from both participants and their parents before testing. All tests were performed indoors in the laboratory. Prior power analysis via G*Power (Version 3.1) determined a required sample size of n = 31 to achieve statistical significance (alpha = 0.05) with 80% power in a linear regression model involving two independent variables (*F* test), assuming a large effect (Cohen’s *f*^2^ = 0.35).

### Design and Procedures

For the jump-testing portion of this study, participants' CMJ performance was assessed using a force plate (type 9260AA6, Kistler, Winterthur, Switzerland) with a sampling rate set at 2500 Hz. Ball velocity during pitching was measured using a pocket radar gun (Pocket Radar Ball Coach, PR1000-BC, Seoul, Republic of South Korea) in km/h. Participants wore indoor sports shoes for both jump and pitching tasks during the study.

All participants were required to complete their own warm-up routine, which included approximately 20 min of whole-body dynamic stretching and pitching drills. After the warm-up, participants wore their own sports shoes and performed three CMJ tests on a force plate. During the CMJ test, we used an external cue such as “Jump as fast and as high as you can upward”. Therefore, participants started in an upright position and quickly squatted down before extending their hip, knee, and ankle joints to jump as high as possible on the force plate. To avoid the influence of arm swinging, participants were asked to keep their hands on their hips throughout the test. After completion of the three CMJ tests, participants were allowed to throw and prepare until they felt comfortable pitching on the mound. Owing to limitations in the laboratory space, an imitation square strike zone (0.4 x 0.5 m) was set eight meters in front of the mound, and participants were required to throw the ball to hit the target. Participants self-reported when they were ready to throw, and we collected five successful pitches. The three fastest velocities were used for further analyses.

### Data Analyses

**Figure 1:**
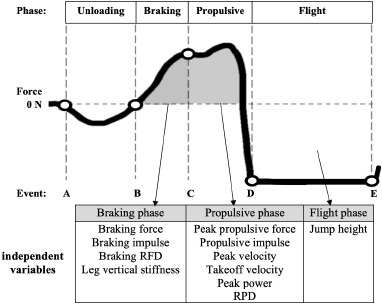
CMJ phase and independent variables. Event: (A) start; (B) minimum negative velocity; (C) zero velocity; (D) takeoff; (E) landing. Abbreviation: RFD = rate of force development, RPD = rate of power development

The ground reaction force (GRF) data collected during the CMJ tests were filtered using a fourth-order low-pass Butterworth filter with a cutoff frequency of 12 Hz. The CMJ takeoff and landing events were determined based on the vertical GRF, with a threshold of 10 N for both events. In addition, the CMJ force variables were derived from the vertical GRF data. Second, using the impulse-momentum method, the net vertical force curve integrated using the trapezoid rule to obtain the time-velocity profile continued calculating the power data. Finally, we integrated the time-velocity data from the initial standing still to landing to obtain the displacement-time data. Therefore, the CMJ movement phases were defined as follows based on the velocity profile: the unloading phase (from the onset of the velocity change to the minimum negative velocity), the braking phase (from the minimum negative velocity to zero velocity, which corresponded to the lowest point of the squat bottom position), the propulsive phase (from zero velocity to takeoff), and the flight phase (from takeoff to landing). All variable analysis formulas for the jump trials are listed in Table 1. In this study, the variables we focused on were mainly from the braking to the flight phase, which including braking force, peak force, the rate of force development (RFD), braking impulse, propulsive impulse, peak concentric power (PP), the rate of power development (RPD), vertical stiffness, peak velocity, takeoff velocity, and jump height (JH). The CMJ movement phases and independent variables used in the analysis are shown in Figure 1.

**Table 1:** Independent variables for CMJ trials.

Variables	Formula
Braking force (N)	The peak vertical GRF applied during the braking phase at the bottom position
Braking impulse (N x s)	Temporal integration of vertical GRF during the braking phase
Braking rate of force development (RFD) (N/s)	Braking force (N) / total time of the braking phase
Peak force (N)	The peak vertical GRF applied during the propulsive phase
Propulsive impulse (N x s)	Temporal integration of GRF from the bottom position to takeoff
Peak concentric power (W)	The peak power during the propulsive phase
Rate of power development (RPD) (W/s)	(Maximum power − minimum power) / total time from maximum to minimum power
Vertical stiffness (N/m)	Braking force / displacement from the start to the bottom position
Peak velocity (m/s)	Peak vertical velocity during the CMJ
Takeoff velocity (m/s)	Vertical velocity at takeoff
Jump height (m)	(Takeoff velocity)^2^ / (2*9.81)

Anthropometric characteristics including body height and mass were used in the analysis. For statistical analysis, the best trial of the three jumps and the average of the top three fastball velocities were used to better assess participants' maximum jumping ability and pitching performance.

### Statistical Analysis

Descriptive statistics were calculated, such as the mean and standard deviation, for all variables, including both absolute and normalized to body mass CMJ data. Normality testing was performed using the Shapiro-Wilk test, and it was determined that all variables were normally distributed. The Pearson’s correlation coefficient and stepwise multiple linear regression analyses were then conducted to investigate the relationships between CMJ variables and fastball velocity in adolescent baseball pitchers, and to determine which combination of variables could predict fastball velocity. Correlation coefficients were classified as high (0.60–0.79), moderate (0.4–0.59), or low (0.2–0.39) based on their *r*-values. Variables with a *p*-value of less than 0.05 were included in the multiple regression analysis, which was conducted in a stepwise manner. All calculations were performed using custom MATLAB script (MATLAB 2022b; MathWorks® Inc., Natick, MA, USA). Statistical analyses were conducted using SPSS version 26.0 (IBM, Chicago, Illinois, USA), with a significance level set at α = 0.05. The results are presented as the mean ± SD.

## Results

**Figure 2:**
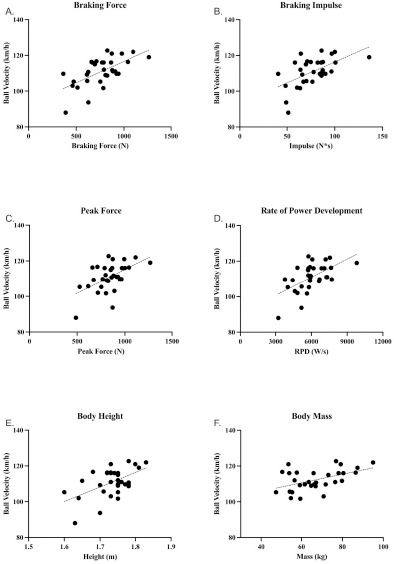
Scatterplots of correlation significant variables (p ≤ 0.001) with ball velocity. A: braking force (R^2^ = 0.38, p < 0.001). B: braking impulse (R^2^ = 0.33, p = 0.001). C: peak force (R^2^ = 0.30, p = 0.001). D: rate of power development (R^2^ = 0.34, p = 0.001). E: body height (R^2^ = 0.29, p = 0.001). F: body mass (R^2^ = 0.32, p = 0.001)

The average fastball velocity was 111 ± 7.8 km/h, ranging from 88 to 122.8 km/h. Table 2 presents the mean ± standard deviation (SD) of absolute and normalized CMJ-derived variables. None of the normalized variables showed any significant association with ball velocity. Table 3 includes the Pearson correlations (r) and significance levels of absolute variables and anthropometrics. This study revealed a total of one high association and seven moderate associations. Scatterplots of the relationship between ball velocity and selected variables are depicted in Figure 2. Table 4 presents the models derived from the regression analysis.

**Table 2:** Descriptive results of original and normalized CMJ variables (*n* = 32).

CMJ Variables (mean ± SD)	Absolute	Normalized (/kg)
Braking force (N)	763.54 ± 202.94	1.18 ± 0.25
Braking impulse (N x s)	77.08 ± 19.12	1.17 ± 0.23
Braking rate of force development (RFD) (N/s)	3978.11 ± 1534.98	60.55 ± 22.66
Peak force (N)	844.32 ± 160.86	1.31 ± 0.17
Propulsive impulse (N x s)	165.37 ± 30.68	2.5 ± 0.19
Peak concentric power (W)	3298.28 ± 619.46	50.13 ± 5.34
Rate of power development (RPD) (W/s)	6005.32 ± 1339.4	91.45 ± 15.79
Vertical stiffness (N/m)	2200 ± 661.94	32.52 ± 8.88
Peak velocity (m/s)	2.7 ± 0.17	-
Takeoff velocity (m/s)	2.5 ± 0.18	-
Jump height (m)	0.32 ± 0.05	-

**Table 3:** Correlations between variables and fastball velocities.

Variables	*r*-value	*p*-value
Braking force (N)	0.618	<0.001†
Braking impulse (N x s)	0.571	0.001†
Braking rate of force development (RFD) (N/s)	0.526	0.002*
Peak force (N)	0.545	0.001†
Propulsive impulse (N x s)	0.537	0.002*
Peak concentric power (W)	0.448	0.01*
Rate of power development (RPD) (W/s)	0.580	0.001†
Vertical stiffness (N/m)	0.510	0.003*
Peak velocity (m/s)	0.062	0.735
Takeoff velocity (m/s)	0.049	0.791
Jump height (m)	0.001	0.995
Body height (m)	0.539	0.001†
Body mass (kg)	0.565	0.001†
* *p* < 0.05, † *p* ≤ 0.001		

**Table 4:** Final models including the regression equation, variance explained (R^2^), and the significance level (*p*), derived from regression analysis to predict fastball velocity.

Variables	*r*	*R*^2^ (adjust *R*^2)^	*p*-value	Regression equation
Braking force (N) (x_1_)	0.618	38.2 (36.1)	< 0.001	y = 0.024x_1_ + 92.78
Braking force (N) (x_1_) + body height (m) (x_2_)	0.686	47.0 (43.4)	< 0.001	y = 0.018x_1_ + 50.2x_2_ + 10.03

## Discussion

This study explored the correlation between the CMJ test, individual characteristics, and pitching velocity in adolescents with the aim of identifying an optimal set of variables for predicting pitching velocity. The results of this study are consistent with our initial hypotheses. Specifically, participants who achieved higher fastball velocities exhibited a significantly greater braking force, impulse, and power during the CMJ test. Conversely, our study found no significant correlation between the jump height and fastball velocity. This suggests a minimal or a negligible relationship between the variables. Given that jump height is modulated by force across different phases of the CMJ (Nishiumi and Hirose, 2024), participants’ jumping heights could not accurately gauge their athletic pitching performance.

To our knowledge, this study represents the first comprehensive investigation of the CMJ force curve and pitching performance in teenagers. Previous research has demonstrated a correlation between various jump tests and ball velocity. Researchers have found that lower-body field test results correlate with the pitching velocity in adults (Huang et al., 2022; Lehman et al., 2013; Lis et al., 2023). Nakata et al. (2013) investigated the relationship between performance variables and anthropometric measurements of baseball ability in youth baseball players and provided evidence (i.e., standing long jump, 10-m sprint, and grip strength) that mirrors the power of the lower body muscle strength as significant predictors of pitched ball kinetic energy. Previous studies often evaluated participants based on jumping distance or height. However, our research applied force plates to obtain more detailed force information, providing deeper insights into the mechanics of jumping to pitching performance.

Among the various CMJ variables investigated, the braking force is one of the most relevant and valuable. This is followed by the rate of power development and braking impulse. However, no significant correlations were found when the body mass was normalized. Previous research has shown that body mass is associated with ball velocity in college level players (Lehman et al., 2013; Werner et al., 2008). As baseball is not a weight-division sport, this may contribute to the limited significance of normalizing the data. We believe this could also be a possible reason why a recent study found no significant correlation between countermovement jump variables and ball velocity when body mass normalized data was used in professional players (Wong et al., 2023). Hence, it is imperative to consider this factor during the analysis of CMJ data. It appears that absolute strength may be more important than relative strength in baseball players.

Recent studies have demonstrated that both braking force and impulse are positively correlated with pitching velocity. Our results indicated that the countermovement jump (CMJ) braking force independently explained 36% of the pitching velocity variance. Considering the concept of the braking phase, the braking force phenomenon in a countermovement jump (CMJ) can be used to assess the power transmission between eccentric and concentric contractions. In contrast, braking impulse can be used to evaluate the overall force output during the braking phase. The primary function of the stride leg is to decelerate the knee flexion, establish a stable foundation for further knee movements, and subsequently transfer power to different phases of the pitching motion (Chu et al., 2016). This transmission is controlled by muscle function during the stretch-shortening cycle and power development efficacy of bodily movements. Moreover, muscular strength of the stride leg contributes to the ability to stabilize and support the body during the dynamic pitching process (Campbell et al., 2010; Matsuo et al., 2001). Therefore, the braking capacity after stride foot contact demonstrated a similar trend to the CMJ’s braking motion: the stride leg underwent continual, greater eccentric force absorption to transfer energy to the body upon foot contact. Given these interactions, it is possible to extract specific information from the CMJ's braking phase of a CMJ. Despite the age groups, recent research has shown that a low eccentric rate of force development in the CMJ’s braking phase is an indicator of high elbow injury risk (Mayberry et al., 2020). Thus, pitchers with better lower extremity strength in CMJ performance could also adequately transfer energy from the kinetic chain to elevate their pitching performance and reduce the potential injury risk.

The findings of our study also highlight the importance of lower-limb power for pitching performance, since the CMJ is a task that mainly relies on lower-limb coordination and explosiveness. The stride leg CMJ’s RPD is positively associated with pitching velocity, which is second only to that of the braking force in this study. After contact, the ground reaction forces exerted by the stride leg in the arm-cocking and arm-acceleration phases are significantly correlated with ball velocity (McNally et al., 2015). During the transition from knee flexion to extension, the knee extensors facilitate the shift from eccentric to concentric contraction, accelerating the muscle power output for follow-up motion to ball release (Campbell et al., 2010). The rapid motion demand for lower-limb power in pitching is similar to the explosive movements of the CMJ, which could explain the rate of power development. Our study findings provide evidence from the CMJ test that a potential correlation exists between enhanced lower-extremity strength and increased pitching velocity, which is consistent with previous research (Guido and Werner, 2012; McNally et al., 2015; Werner et al., 2008; Wong et al., 2023). Consequently, we propose that the CMJ test has the potential to provide valuable insight into the force output and functionality of the stride leg. It is possible to customize training programs for pitchers to enhance jumping-related process variables, thereby resulting in increased lower-extremity power and braking ability. These insights could be beneficial for enhancing force transfer and assisting pitchers in attaining the highest pitching velocity.

The regression analysis revealed that the combination of the absolute braking force and body height was significantly associated with ball velocity, explaining approximately 43% of its variance. Linear regression further allowed us to extrapolate and state that when players had the same absolute braking force, the fastball velocity increases by 5 km/h for every 10-cm increase in body height. Similarly, at the same height, the fastball velocity increased by 1.8 km/h for every 100-N increase in body mass. This interpretation suggests that pitchers with greater absolute braking force during the CMJ and a taller body height at the same time will have a better probability of advancing their fastball velocity at the youth stage. Previous studies have indicated a relationship between body height and pitching performance (Nakata et al., 2013; Tremblay et al., 2022). This may be due to the fact that taller pitchers can generate more leverage and power to transmit force onto the ball (Sgroi et al., 2015).

However, whether this relationship holds in adult populations (e.g., collegiate or professional players) remains uncertain. While anthropometric factors such as body height and body mass remain relevant to performance, their predictive power may differ as pitchers mature and performance becomes increasingly influenced by refined motor patterns, technical skill, and accumulated training experience. For example, Huang et al. (2024) reported that the CMJ and sprint ability, along with body height, significantly contributed to pitching velocity in adult pitchers; however, these variables explained only 23% of the variance. In contrast, factors such as pitching biomechanical efficiency, muscle strength, and neuromuscular control may play a more dominant role in adults. Notably, increased body mass in adulthood may enhance the total energy transferred to the ball. Werner et al. (2008) found that body mass combined with pitching kinematic variables explained up to 68% of the variance in ball velocity among collegiate pitchers. Moreover, the direction and plane of movement used to generate power during pitching also merit consideration. Lehman et al. (2013) demonstrated that a greater distance in lateral-to-medial jump performance, when combined with increased body mass, may account for greater kinetic energy directed toward the target, thereby increasing ball velocity in collegiate pitchers. These findings suggest that adult pitching performance is influenced by more complex and integrated mechanisms of energy transfer and mechanical efficiency. In contrast, youth pitchers are still developing technical proficiency, and thus, the ability to brake and generate force effectively after a stride may play a more pivotal role during their pitching. Our study showed that CMJ force performance might reflect this braking capacity, providing valuable insight at this developmental stage. While the present findings are informative for identifying key factors associated with ball velocity in youth players, caution is warranted when generalizing these results to adult populations.

In addition, research in various sports has revealed that in youth athletes, sports performance is influenced more by the state of maturation and physical changes (Velez et al., 2025) than by age (Perroni et al., 2024). Significant improvements in the performance of youth athletes frequently occur during the period of their growth peak, when adolescents experience the greatest growth rate in height (Philippaerts et al., 2006). The data suggest that maximal gains in muscular strength and power occur after peak height velocity in adolescent males (Beunen and Malina, 1988). Players can vary significantly in terms of the pace of physical development at the teenage level. Therefore, within the context of aptitude identification and development, coaches and trainers should be well informed about the aspects of adolescent growth that can impact both their jumping force output and pitching performance. Nonetheless, we suggest that enhanced braking ability and physical development may contribute to enhanced pitching velocity in adolescents. The lower extremity strength and functional ability may be effectively evaluated through analysis of the GRF components during CMJ testing.

Several limitations of this study must be acknowledged. First, although we showed that lower limb strength measured by the CMJ test was associated with ball velocity during pitching motion, this is only one part of the pitching kinematic chain that can influence speed. Other pitching biomechanical metrics should be considered in future studies to explain the variance in these models better. Second, our performance findings may only apply to pitchers with a certain level of pitching experience. The current data were collected from a cohort of adolescent junior high school pitchers, which limits our ability to generalize the results to pitchers of different ages and skill levels. Finally, our assessment was limited to a combined analysis of both legs on a single force plate. However, recent research has examined the impact of bilateral and unilateral jumps on collegiate baseball pitchers (Lis et al., 2023). Further studies should consider this aspect of jump tests among adolescents. In future research, these variables should be thoroughly investigated to better understand the correlation between countermovement jump performance, pitching GRF, and ball velocity in the context of baseball pitching.

## Conclusions

In summary, the key findings of the current study emphasize the crucial role of lower limb strength in adolescent pitchers, and we suggest that CMJ data can help evaluate throwing velocity ability. We found that certain absolute force-related variables from the CMJ test and physical stature significantly correlated with pitching performance. These correlations could impact ball velocity among adolescent players, with moderate to strong correlations. Pitchers with a higher absolute braking force during the CMJ and a taller body have a better chance of increasing their fastball velocity. Therefore, our findings support the importance of lower-limb strength for pitching performance, and these characteristics can be considered when selecting pitchers during off-season physical measurements. Our study provides valuable insights into these relationships, which could help coaches evaluate pitching aptitude, develop training programs, and enhance performance of teenagers. We recommend that adolescent pitchers engage in lower limb strength training to enhance their potential fastball velocity.
